# 

**Published:** 1880-03-20

**Authors:** 


### *THE INDEX MEDICUS.*

We notice in the announcement of the above journal for 1880 that the editors have encountered heavy difficulties in entering upon the second year of its publication. This journal should be encouraged by the medical press and sustained by the profession throughout the country.

### *MEDICAL ASSOCIATIONS.*

The Medical Association of the State of Georgia will convene in Augusta April 21st.

The Kentucky Medical Society meets in Lexington on the first Tuesday in April next.

The American Medical Association meets in New York City on the first Tuesday in June next.

### *RECEIPTED.*

[Receipts not acknowledged privately are entered here.]

1880 - J. W. Sanders, John Durham, J. C. Boozer, R. J. Talbot, T. E. Jennings, J. H. Jennings, J. T. Davis, W. B. Sikes, W. C. Jones, J. S. Horseley, W. T. Mathes, P. Taylor, J. B. Bailey, T. L. Quillian, P. M. Catching, J. S. Green, V. J. Smith, A. H. Sellers, A. J. Woolverton, L. W. Mobley, S. M. Hogan, W K. Jones, J. A. Hays, B. F. Darnell, W. S. Harris, Lanier & McCabe, L. B. Bouchelle, P. P. Terry, J. F. Pou, J. O. Sanders, W. H. Stewart, Louis Hadden, S. W. Eaton, F. M. Rushing, J. R. Johnson, J. D. Harrell. 1879—A. B. Loving, M. B Pollard, W Barton, W. P. Anderson, E. L. Dodd, B. Dunnahoe, T. A. Mattox, B. B. Smiley, E. O. Tucker, R. Winthrop, J. Bonner, L. L Smythe.

